# Extracellular Vesicles in Hematological Disorders

**DOI:** 10.5041/RMMJ.10166

**Published:** 2014-10-29

**Authors:** Anat Aharon, Annie Rebibo-Sabbah, Inna Tzoran, Carina Levin

**Affiliations:** 1 Microvesicles Research Laboratory, Thrombosis and Hemostasis Unit, Department of Hematology, Rambam Health Care Campus;; 2 Bruce Rappaport Faculty of Medicine, Technion, Israel Institute of Technology, Haifa, Israel;; 3 Department of Internal Medicine C, Rambam Health Care Campus, Haifa, Israel; 4 Pediatric Hematology Unit and Pediatric Department B, Emek Medical Center, Afula, Israel.

**Keywords:** Extracellular vesicles, exosomes, microRNA, thrombogenicity, hemoglobinopathies, leukemia, myeloma, lymphomas

## Abstract

Extracellular vesicles (EVs), comprised of exosomes, microparticles, apoptotic bodies, and other microvesicles, are shed from a variety of cells upon cell activation or apoptosis. EVs promote clot formation, mediate pro-inflammatory processes, transfer proteins and miRNA to cells, and induce cell signaling that regulates cell differentiation, proliferation, migration, invasion, and apoptosis. This paper will review the contribution of EVs in hematological disorders, including hemoglobinopathies (sickle cell disease, thalassemia), paroxysmal nocturnal hemoglobinuria, and hematological malignancies (lymphomas, myelomas, and acute and chronic leukemias).

## INTRODUCTION

**Figure 1.**
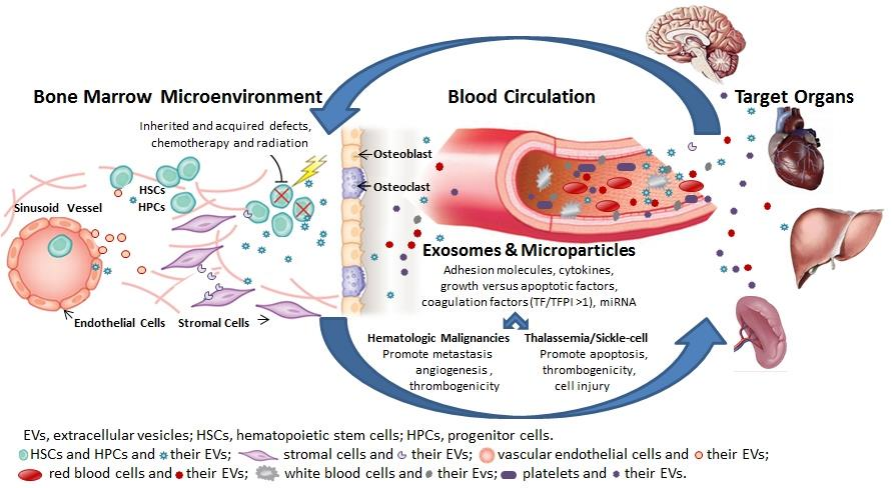
**Involvement of Extracellular Vesicles (EVs) in Hematologic Disorders.** Inherited and acquired defects as well as exposure to chemotherapy, radiation, and cytokines result in release of EVs from a variety of cells (e.g. hematopoietic stem and progenitor cells, blood, vascular, and tumor cells) to the bone marrow microenvironment, the vascular compartment, and the target organ with auto- and paracrine effects. Extracellular vesicles, which include microparticles and exosomes, express adhesion molecules, cytokines, growth versus apoptotic factors, coagulation factors and miRNA. In hematologic malignancies, EVs promote metastasis, angiogenesis, and thrombogenicity. In thalassemia/sickle cell diseases, EVs promote cell injury, apoptosis, and thrombogenicity.

There are millions of extracellular vesicles (EVs) in the circulation of healthy persons, and their level may increase in a variety of pathologies. EVs may be divided into sub-groups, i.e. exosomes, micro-particles, and apoptotic bodies, which are shed from both normal and malignant cells upon cell activation or apoptosis. Extracellular vesicles promote clot formation, mediate pro-inflammatory processes, facilitate cell-to-cell interactions, transfer proteins and miRNA to cells, and induce cell signaling (Figure 1). This paper will review earlier studies which focus on the role of EVs in hematological disorders, including hemoglobinopathies (sickle cell disease, thalassemia), paroxysmal nocturnal hemoglobinuria, and hematological malignancies (lymphomas, myelomas, acute and chronic leukemias). In addition, it will review the involvement of EVs in the hypercoagulability characterizing these hematological disorders.

## EXTRACELLULAR VESICLES

Extracellular vesicles are detectable in the blood under normal physiological conditions, where their prevalence is increased in cases of cancer, inflammation, cardiovascular disease, diabetes, and preeclampsia.^1^ Extracellular vesicles, a wide variety of microvesicles including exosomes and microparticles, are shed from normal cells (platelets, endothelial cells, leukocytes, and monocytes) as well as from malignant cells. Exosomes are intracellular luminal vesicles (50–90 nm), originating from endosomes, that fuse with the cell plasma membrane and release their content to the cell surroundings and blood circulation. The composition of exosomal surface proteins enables tracing of their specific endosomal origin.^2^ Exosomes include diverse subpopulations of vesicles secreted via different intracellular mechanisms,^3^ and proteins of endosomal sorting complexes required for transport (ESCRT) machinery play a role in their biogenesis.^4^,^5^ Microparticles are membrane vesicles (∼1 µm in diameter) shed from the cell surface to the cell surroundings and blood circulation following stimulation, and are often a hallmark of cell apoptosis. Microparticle membrane proteins reflect those of the origin cell. Overall, EVs modulate target cells by transferring proteins and miRNA to neighboring cells, elevating protein expression on the target cell membrane, and inducing cell signaling pathways that affect cell functions including differentiation, proliferation, migration, invasion, and apoptosis.^6^–^9^ In addition, microparticles bearing coagulation and adhesion molecules, such as P-selectin and P-selectin glycoprotein ligand-1 (PSGL1), play a central role in coagulation initiation and thrombus formation.^10^ Cell exposure to cytokines (tumor necrosis factor-alpha and interleukin-1) or chemotherapy results in microvesicle secretion.^11^ Chemotherapy leads to platelet activation and generation of EVs associated with increased risk of thrombosis.^12^ Additional triggers, such as complement cascade proteins,^13^ states of hypoxia, irradiation, oxidative injury, or shear stress, increase the number of EVs shed by cells.

## EXTRACELLULAR VESICLES AND THROMBOGENICITY

Extracellular vesicle-associated tissue factor (TF), the main activator of the coagulation cascade, plays a major role in pathogenesis of the prothrombotic state observed in cancer patients.^14^ Tissue factor is expressed on non-vascular cells, activated cells within the vessel wall (such as leukocytes and endothelial cells), and circulating EVs.

A model defining the role of hemostatic versus pathological microparticles in the mechanism of thrombus formation was recently proposed.^15^ In healthy people, despite the appearance of circulation of TF-bearing EVs, the TF on the EVs is in an inactive form; it is activated upon recruitment to a site of vascular injury. However, in pathological states, EVs derived from tumor or inflammatory cells bear an active form of TF that can induce thrombotic events *in vivo*. Tumor cells express TF that spontaneously release TF-positive EVs, exhibit strong procoagulant activity, and are the main initiators of thrombus formation,^16^,^17^ which may explain the increased rates of venous thrombosis in patients with cancer.^18^ Therefore, circulating EVs bearing active TF and especially the ratio between TF and its inhibitor tissue factor pathway inhibitor (TFPI) on EVs have the potential to be an indicator of a hypercoagulable state in cancer patients and can serve as potential biomarkers of individuals with an increased thrombotic risk.^19^ The procoagulant properties of EVs may also be attributed to the high level of phosphatidylserine, which provides a catalytic site for coagulation complexes such as prothrombinase and tenase, thereby indirectly enhancing coagulation activation.^20^ Hence, the negative charge of the EVs leaflet is a key factor in coagulation processes. In addition, the phosphatidylserine leaflet negative charge promotes membrane fusion and transfer of both proteins and lipids to other cells.^21^

## EXTRACELLULAR VESICLES IN HEMOGLOBINOPATHIES

Sickle cell disease and β-thalassemia represent the most common hemoglobinopathies caused, respectively, by the alteration of structural features or deficient production of the β-chain of the hemoglobin molecule. In addition there is increasing evidence that these hemoglobinopathies are also associated with a state of chronic hypercoagulability.

### Sickle Cell Disease EVs

Sickle cell disease is a chronic, inflammatory disease resulting from hemoglobin-S-related chronic hemolysis, a microvascular circulatory disorder with recurrent vascular occlusions triggered by red blood cell and leukocyte adhesion to the vascular endothelium. The disease is associated with a hypercoagulable and pro-inflammatory state as well as endothelial dysfunction.^22^

In transgenic SAD mice (a model which expresses a modified sickle hemoglobin, Hb SAD, and displays *in vivo* hemoglobin polymerization and erythrocyte sickling), thrombospondin-1 triggers erythrocyte microparticle shedding. These microparticles induce endothelial injury and facilitate acute vaso-occlusive events.^23^ In humans, microparticles also seem to be involved in the pathogenesis of sickle cell disease. Microparticle levels in patients with sickle cell disease were found to be significantly higher during both painful crisis and steady-state situations compared with the control group. Their level did not reflect the high frequency of crisis. A higher concentration of platelet-derived microparticles was detected in patients with severe sickle cell anemia (SCA) and nephropathy, while erythrocyte-derived microparticle concentration was increased in the non-severe SCA patient group and during crisis compared to the steady-state period.^24^,^25^ In children, both platelet and erythrocyte microparticle levels were positively correlated with aortic stiffness and pulmonary artery pressure, while they were found to correlate negatively with aortic distensibility.^25^ Nébor et al. reported hydroxyurea treatment to be associated with a decrease in microparticles derived from erythrocytes and platelets,^26^ whereas in another study endothelial microparticle levels were not affected by hydroxyurea treatment.^22^

### Thalassemia EVs

Thalassemia is a group of inherited hemoglobinopathies leading to reduced or absent synthesis of alpha or beta globin chains and resulting in variable outcomes ranging from severe anemia to clinically asymptomatic states. The mutations disrupt normal production of hemoglobin and cause low hemoglobin levels and a high rate of red blood cell destruction, causing anemia. The complications include bone changes, hypercoagulability, and end-organ damage due to iron overload.^27^

Several studies have evaluated and characterized the microparticles in β-thalassemia syndromes. These studies focused on characterization of microparticle numbers, their cell origin, and thrombogenicity. Thalassemia patients have a significantly higher level of microparticles than normal subjects. These microparticles mostly originate from platelets and red blood cells and express negatively charged phospholipids (PS).^28^ The number of platelet microparticles was significantly higher in splenectomized versus non-splenectomized patients, and levels of procoagulant microparticles of red blood cell, leukocytic, and endothelial origins were higher in patients with β-thalassemia intermedia than in controls. These higher levels may have the potential to aggravate thrombotic events.^29^ High levels of microparticles correlated with the increased procoagulant activity and with the increased platelet counts of the patients. Another study found significantly elevated microparticle levels in thalassemic patients compared with controls, particularly in patients with a risk of pulmonary hypertension, history of thrombosis, splenectomy, and high serum ferritin levels. This increase in microparticles may be implicated in vascular dysfunction, pulmonary hypertension risk, and aortic wall stiffness observed in thalassemia patients.^25^ Positive correlation was found in thalassemia intermedia patients between the amount of hemichromes measured in erythrocytes, their capability to release microparticles, and the levels of plasma hemichromes.^30^

## PAROXYSMAL NOCTURNAL HEMOGLOBINURIA AND EVS

Paroxysmal nocturnal hemoglobinuria (PNH) is a rare acquired stem cell disorder caused by a somatic mutation of phosphatidylinositol glycan A. As a consequence, membrane inhibitors of complement are lost, rendering the cells more susceptible to complement-mediated destruction leading to diverse clinical manifestations which include intravascular hemolysis, bone marrow failure, and significant thrombosis (thromboembolism accounts for approximately 40% to 67% of deaths). Paroxysmal nocturnal hemoglobinuria can be “primary” or “secondary” in the context of other bone marrow disorders such as aplastic anemia. Several factors are involved in thrombogenesis in PNH: (1) chronic hemolysis, (2) impaired fibrinolytic system, (3) microparticles released from injured platelets and vascular endothelial cells.^31^ Platelet activation in PNH patients is accompanied with phosphatidylserine externalization, a key process in shedding of microparticles.^32^ Paroxysmal nocturnal hemoglobinuria patients had elevated counts of platelet and endothelial microparticles with prothrombotic and pro-inflammatory phenotypes. Circulating microparticles of PNH patients express endothelial markers: intercellular adhesion molecule 1 (ICAM-1), soluble vascular cell adhesion molecule-1 (sVCAM-1), von Willebrand factor, CD144 (VE-cadherin), indicating chronic endothelial activation and vascular inflammation.^33^,^34^ Eculizumab (a monoclonal antibody to complement protein 5) therapy results in rapid and sustained decreases in markers of inflammation, thrombin generation, and TF-bearing microparticles in PNH patients.^35^

## EXTRACELLULAR VESICLES AND CANCER

Several reviews have summarized the involvement of EVs in cancer. Tumor cells express TF, and cancer microvesicle-barring TF trigger the coagulation cascade associated with high risk for thrombotic events.^21^,^36^ In addition, chemotherapy leads to platelet activation and generation of microparticles, associated with increased risk of thrombosis.^12^ Additional triggers, such as complement cascade proteins^13^ or coagulation-related thrombin expression,^11^ as well as states of hypoxia, irradiation, oxidative injury, or shear stress, increase the number of procoagulant EVs shed by malignant and non-malignant cells.

Tumor EVs are involved in tumor progression and regulate tumor cell proliferation. Extracellular vesicles contain growth factors and cytokines that support angiogenesis and formation of new blood vessels at the tumor microenvironment, which are critical for sustaining tumor growth. Extracellular vesicles were found to modulate immune responses, affect oncogenic signaling pathways, and promote tumor metastasis.^37^,^38^ Development of multi-drug resistance remains a key factor of cancer treatment failure in the majority of patients with metastatic disease. Extracellular vesicles mediate transfer of multidrug resistance-associated proteins, which affects the intrinsic resistance pathways of the recipient cells.^39^,^40^

Circulating EVs also serve as transport vehicles for large numbers of specific microRNAs (miRNAs) and have been associated with vascular diseases.^41^ MicroRNAs are ∼22-nt long non-coding RNAs that play an important role in gene regulation by binding to mRNA at their complementary sequence. MicroRNAs affect key biological processes, such as cell growth, tissue differentiation, cell proliferation, and apoptosis.^42^ MicroRNAs are connected with various signal transduction pathways regulating diseases, including hematological malignancies, and they provide new potent markers for efficient diagnosis and prognosis for patients with hematological malignancies. MicroRNA profiles of EVs are significantly different from their maternal cells, indicating an active mechanism of selective “packaging” and delivery from one cell to another.^43^ There are several miRNA that are involved in regulation of normal hematopoiesis, including miR-17, miR-24, miR-146, miR-155, miR-128, and miR-181, which prevent the differentiation of early-stage progenitor cells, while miR-16, miR-103, and miR-107 act later on, and miR-221, miR-222, and miR-223 control the terminal stages of hematopoietic development.^44^ The general importance of miRNA dysregulation for the pathogenesis of myeloid disorders is explained by the fact that more than 70% of all human miRNAs are encoded within regions of recurrent copy-number alterations in myelodysplastic syndrome (MDS) and acute myeloid leukemia (AML) cell lines. Furthermore, a functional role in the pathology of these diseases has been demonstrated recently by several *in vivo* mouse models. In addition, numerous studies have defined miRNA signatures associated with chronic lymphocytic leukemia (CLL) diagnosis and prognosis that could distinguish between the aggressive and the indolent form. Diffuse large B-cell lymphoma (DLBCL) was one of the first lymphomas to be linked with aberrant miRNA expression, in particular with over-expression of miR-155.

Finally, acute graft-versus-host disease (aGVHD) is the major cause of early morbidity and mortality in allogeneic hematopoietic stem cell transplantation recipients.

Significantly higher levels of endothelial microparticles were detected in the patients during aGVHD that might indicate severe endothelial cell injury,^45^ and there was a significant difference in endothelial microparticle levels between aGVHD group and a non-aGVHD group.^46^ In addition, increased plasma levels of erythrocyte-derived microparticles were found in patients who developed aGVHD but not in patients who developed infection or sepsis after hematopoietic stem cell transplantation.^47^

## HEMATOLOGICAL MALIGNANCIES AND EXTRACELLULAR VESICLES

“Hematological malignancies” is a broad term that includes blood cell cancers, i.e. CLL, chronic myeloid leukemia (CML), AML, myelodysplastic syndrome, acute lymphocytic leukemia, multiple myelomas (MM), and lymphomas. The involvement of EVs in hematological malignancies has been poorly investigated, and the data on EV characterization and their effects are limited.

### Chronic Lymphocytic Leukemia EVs

B-cell chronic lymphocytic leukemia (B-CLL) has been predominantly characterized as a clonal B-cell disorder in which the defective apoptosis of CLL B-cells is ascribed not only to intrinsic defects of neoplastic cells but also to extrinsic factors that influence their behavior in the tissue microenvironment. The CLL-B-cells of most patients express a constitutively active receptor tyrosine kinase (RTK) AXL that was found to be important for CLL B-cell survival. It acts as a docking site and regulates activation of non-receptor kinases, including Lyn and phosphatidylinositol 3-kinase/AKT. Plasmatic microvesicles of CLL patients that originated from B-cells carry a constitutively phosphorylated RTK AXL.^48^ Microvesicles circulating in plasma of B-cell CLL patients exhibit a phenotypic shift from predominantly platelet-derived microvesicles, at an early stage, to leukemic B-cell-derived ones at an advanced stage. Furthermore, the total EV level in CLL was significantly greater than in healthy subjects. The CLL-microvesicles can activate the AKT target of rapamycin/p70S6K/hypoxia-inducible factor-1α axis in CLL bone marrow stromal cells (BMSCs) with production of vascular endothelial growth factor, a survival factor for CLL B-cells. Moreover, EV-mediated AKT activation led to modulation of the β-catenin pathway and increased expression of cyclin D1 and c-myc in BMSCs.^49^

### Chronic Myeloid Leukemia EVs

Chronic myeloid leukemia (CML) is a clonal myeloproliferative disorder characterized by the presence of Philadelphia chromosome, encoding the chimeric Bcr–Abl onco-protein with constitutive tyrosine kinase activity. A recent study found that exosomes released from CML cells stimulate bone marrow stromal cells to produce interleukin (IL)-8 (mRNA and protein), a potent pro-angiogenic factor that modulates both *in vitro* and *in vivo* the leukemia cell malignant phenotype.^50^ Exosomes derived from human K562 CML cells induce angiogenic activity in HUVEC and in a mouse matrigel plug model of angiogenesis.^51^ Exosomes derived from LAMA84 CML-cells induce an increase in the level of both ICAM-1 and VCAM-1 cell adhesion molecules and IL-8 expression on HUVEC and promote cell migration.^52^ Exosomes obtained from the K562 CML cell line were internalized by endothelial cells and induced tube formation through the activation of Src signaling that linked to cancer cells.^51^ The gene BCR/ABL DNAs that was transferred from injected K562 CML EVs induced partial CML characteristics in rats, 2 months after administration.^53^

### Acute Myeloid Leukemia EVs

Acute myeloid leukemia is characterized by rapid growth of abnormal blast cells that accumulate in the bone marrow and interfere with the production of normal blood cells. Bone marrow samples at diagnosis display enhanced angiogenesis and increased vascular endothelial growth factor A (VEGFA) expression.^54^ Patients with AML can develop venous thromboembolism despite thrombocytopenia.^55^ One case report related to AML EVs found that in two of three AML patients before chemotherapy the high number of circulating leukemic cells correlated with a relatively low percentage of platelet-derived microparticles.^56^

### Multiple Myelomas and EVs

Multiple myeloma (MM) is a B-cell malignancy characterized by monoclonal proliferation of plasma cells in the local bone marrow environment and the development of osteolytic bone lesions. The biology of malignant plasma cells in MM is highly influenced by the bone marrow microenvironment in which they reside, and bone marrow stromal cells are known to interact with MM cells to promote MM cell survival and proliferation.^57^

A study in a long-term MM mouse model found a significant increase of the total circulating micro-particles and bone marrow microparticles compared to controls. Circulating microparticles mainly originated from leukocyte and erythrocyte cells, while EVs of the bone marrow expressed the plasma cell marker CD138 (syndecan-1).^58^

A recent *in vitro* study found MM EVs isolated from the MM cell line (RPMI 8226) transferred CD138 to the endothelial cells and significantly induced cell proliferation, invasion, tube formation, and secretion of IL-6 and VEGF, key angiogenic factors of myeloma. MM cell-derived EVs obtained from patient MM cells and human MM cell lines (HMCLs) were found to be enriched with CD147, a transmembrane molecule crucial for MM cell proliferation, stimulating MM cell growth and enhancing tumor cell proliferation.^59^

## IN SUMMARY

Microvesicles, which include microparticles and exosomes, serve as a “vehicle” or transporter that carries regulatory molecules between cells in physiologic and pathologic states and therefore play a crucial role in thrombosis, inflammation, angiogenesis, and vascular dysfunction that affects the prognosis of a variety of hematologic disorders. The hypercoagulable state related to hematologic disorders may be partially influenced by an increase in the total number of EVs, part of which expressed negatively charged phospholipids. Additionally, the balance between pro- and anti-coagulant factors on EVs shifts to hypercoagulability and may result in thrombotic events. Extracellular vesicles are also involved in angiogenic processes that may play a significant role in the bone marrow microenvironment communication and may affect cell survival/apoptosis in distant organs such as spleen and liver in hematologic malignancies. These EVs also affect metastatic processes and development of multidrug resistance.

The data on the involvement of EVs in hematologic disorders are still limited, and further studies are warranted.

## Abbreviations:

aGVHD: acute graft-versus-host disease;
AML: acute myeloid leukemia;
BMSCS: bone marrow stromal cells;
CLL: chronic lymphocytic leukemia;
DLBCL: diffuse large B-cell lymphoma;
EVs: extracellular vesicles;
ICAM-1: intercellular adhesion molecule 1;
IL: interleukin;
MDS: myelodysplastic syndrome;
miRNAs: miR, microRNAs;
MM: multiple myeloma;
PNH: paroxysmal nocturnal hemoglobinuria;
PSGL1: P-selectin glycoprotein ligand-1;
SCA: sickle cell anemia;
SVCAM-1: soluble vascular cell adhesion molecule-1;
TF: tissue factor;
TFPI: tissue factor pathway inhibitor.
